# New Techniques for Assessing Critical Raw Material Aspects in Energy and Other Technologies

**DOI:** 10.1021/acs.est.2c05308

**Published:** 2022-11-24

**Authors:** Nick Martin, Cristina Madrid-López, Gara Villalba-Méndez, Laura Talens-Peiró

**Affiliations:** † Sostenipra Research Group, Institute of Environmental Science and Technology (ICTA-UAB), Autonomous University of Barcelona, Bellaterra (Cerdanyola del Vallès), Barcelona, Catalunya08193, Spain; ‡ Department of Chemical, Biological and Environmental Engineering, Autonomous University of Barcelona, Bellaterra (Cerdanyola del Vallès), Barcelona, Catalunya08193, Spain

**Keywords:** energy transition, critical raw materials, material supply, life cycle assessment, just transition, energy justice, energy modeling

## Abstract

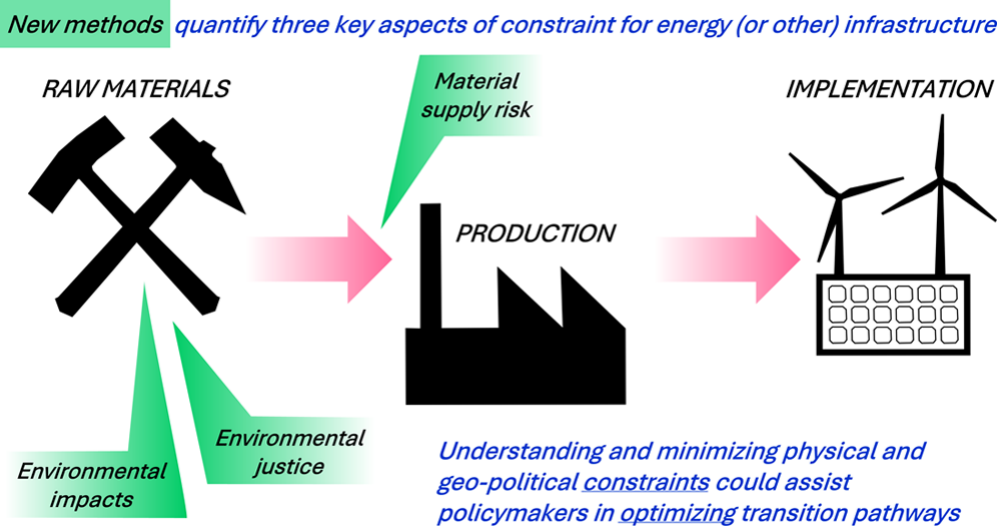

Transitioning to more sustainable energy technologies is a vital step in the move toward reducing global greenhouse gas emissions. However, several physical constraints could hinder the implementation of these technologies, and many of the raw materials required to produce new infrastructure are scarce, nonrenewable, and nonsubstitutable. Various factors relating to material extraction and processing activities may also affect the security and sociopolitical aspects of future supply lines. Here, we introduce methods for quantifying three key indicators relating to raw material supplies for specific production processes: (1) overall supply risk, (2) environmental impacts from sourcing raw materials, and (3) environmental justice threats at sourcing locations. The use of the proposed methods is demonstrated via an exploratory case study examining projected electricity production scenarios within the European Union. Results suggest that renewable sources of electricity—particularly wind, solar, and geothermal technologies—are more likely to exacerbate supply risks and environmental issues than other technologies. Furthermore, projected expansions of wind and solar technologies mean that all three indicators appear likely to rise significantly systemwide by 2050. Ultimately, the methods represent a much-needed first attempt at providing practitioners with simple and robust approaches for integrating factors relating specifically to raw material supply into energy modeling and other applications.

## Abstract 2

Various physical and geopolitical factors relating to raw material supply could constrain the renewable energy transition. This study introduces methods that utilize life cycle material requirement data to quantify three such factors

## Introduction

Scientists and policymakers have now widely accepted the need to reduce emissions of greenhouse gases (GHGs) at all scales.^1^ This is reflected in symbolic global initiatives like the Paris Agreement^2^ and in the many national, regional, and local policies that are being formulated to address the issue. Within the rapidly evolving arena of energy and environmental policy, the need to accelerate the adoption of more “sustainable” sources of energy is viewed as one of the key pathways to reducing emissions and achieving future targets.^3^

However, the concept of sustainability in energy systems is evolving beyond the mere reduction of GHG emissions. Among other things, the ongoing sourcing of the raw materials and components required to implement new infrastructure continues to gain policy focus^4−8^ and mainstream media attention,^9−13^ and several potential “roadblocks” have been identified. The range of issues triggered by the COVID-19 pandemic and war in Ukraine has further highlighted the vulnerability of infrastructure development to supply chain disruptions.^14,15^

A number of specific concerns have been raised in this regard, mostly surrounding the available stocks of necessary materials,^16−18^ geopolitical and governance issues associated with supplying countries,^19−21^ and the issues of social justice and localized environmental damages that surround the increased demand for materials.^22−24^ All three aspects are likely to play a role in determining the speed and direction of the energy transition going forward. The European Commission (EC) has begun to quantify supply risk for specific materials^16^ and now includes geographical concentration and governance, import reliance, and responsible sourcing aspects as part of its triennial “Raw Materials Scoreboard” assessments.^25^ A handful of additional studies have also attempted to measure other aspects of material sourcing, particularly in relation to justice and conflict issues.^26−28^ However, these assessments generally only apply to individual materials. As such, despite a relative paucity of suitable data, a clear need for the quantification of raw material-related constraints relating to individual technologies and processes is arising, particularly for those attempting to optimize systemwide transition pathways and minimize the exposure of these pathways to risk.

To bridge this gap, we present here a series of methods—developed for assessing energy system characteristics as part of the SENTINEL project^29^—that use raw material inventory information from life cycle assessment (LCA) databases alongside other data sources to generate three unique indicators specifically related to the supply of raw materials. First, the risk of interruption to raw material supply channels is quantified by incorporating supply risk data published by the EC.^16^ Two further indicators attempt to quantify the possibility of localized issues occurring during the extraction and processing of raw materials: the potential to exacerbate local environmental conditions is estimated using ecosystem and human health data relating to individual materials from the Ecoinvent LCA database,^30,31^ while the potential to reproduce local environmental justice issues is quantified using data relating to sourcing countries within the Worldwide Governance Indicators (WGI) data set.^32,33^ Collectively, we believe these three indicators represent the majority of key issues in relation to raw material supply at present.

The methods enable composite values to be derived for individual technology types or, indeed, for any unit process defined within LCA databases; higher scores highlight processes that involve material sourcing from locations with higher inherent risks of supply interruption, with poor environmental impact characteristics, or where environmental justice issues are potentially more likely to occur. Values could be integrated into existing energy modeling applications to account for these aspects—for example, as in-built calculations or soft-linked constraint parameters within integrated assessment models or other energy system models—or be used as standalone indicators for assessing proposed energy system configurations in other applications. Full descriptions of the methods and suggested data inputs for each indicator are provided. The approach is then operationalized via a case study involving current and projected scenarios for the European Union (EU) electricity network. A validation and sensitivity analysis is also provided. Findings from the case study and further aspects of the methods are then discussed alongside a final set of conclusions.

## Methodology

The proposed methods all use material requirement information provided by life cycle inventory (LCI) data as their foundation. An LCI represents one of the four phases within a life cycle assessment (LCA).^34^ During this phase, all of the elementary material and energy flows that occur within a process are determined. This includes all sub-processes that occur during the material extraction, processing and manufacturing stages—and, if required, the product use and disposal stages—within the entire life cycle of a process. The resulting breakdown includes listings of all inputs and outputs that occur for a range of different materials. Furthermore, it will include specific items for the process in question—the “foreground” system—alongside those for the broader industrial economy—the “background” system. Final material requirements are given as the total mass of a material required to produce one “unit” of a process. Here, we use a small selection of the available LCI data—using the Ecoinvent database^30^—to perform a customized set of calculations relating to the supply of a specific set of raw materials to a given process.

The methods use 55 of the raw materials identified as being most important to the EU in accordance with the latest list of so-called critical raw materials (CRMs) published by the EC. The most recent investigation, from 2020,^16^ considers a group of 80 materials as potential CRM candidates, of which 44 were deemed critical using a standardized methodology^35^ based on economic importance and supply risk factors. The list includes the five platinum group metals, 10 heavy rare earth, and five light rare earth elements; holmium, thulium, lutetium, and ytterbium are grouped as a single heavy rare earth entry.

An attempt to align the 80 candidate materials from the 2020 EC study with the listings in the December 2020 version of the Ecoinvent LCI database^30^ found that 30 of the 44 CRMs and 25 of the remaining 36 candidate materials are represented in the database; full documentation is provided in the Supporting Information. While it is observed that 25 of the 80 materials were found to have no suitable match in the LCI database, it is noted that 14 of these “missing” materials were categorized as “industrial and construction”—for example, aggregates, rocks, sand—or “biological and other”—for example, rubber, cork, and wood—many of which are either too generic, not relevant, or too complex to quantify in LCI listings.

**Figure 1:**
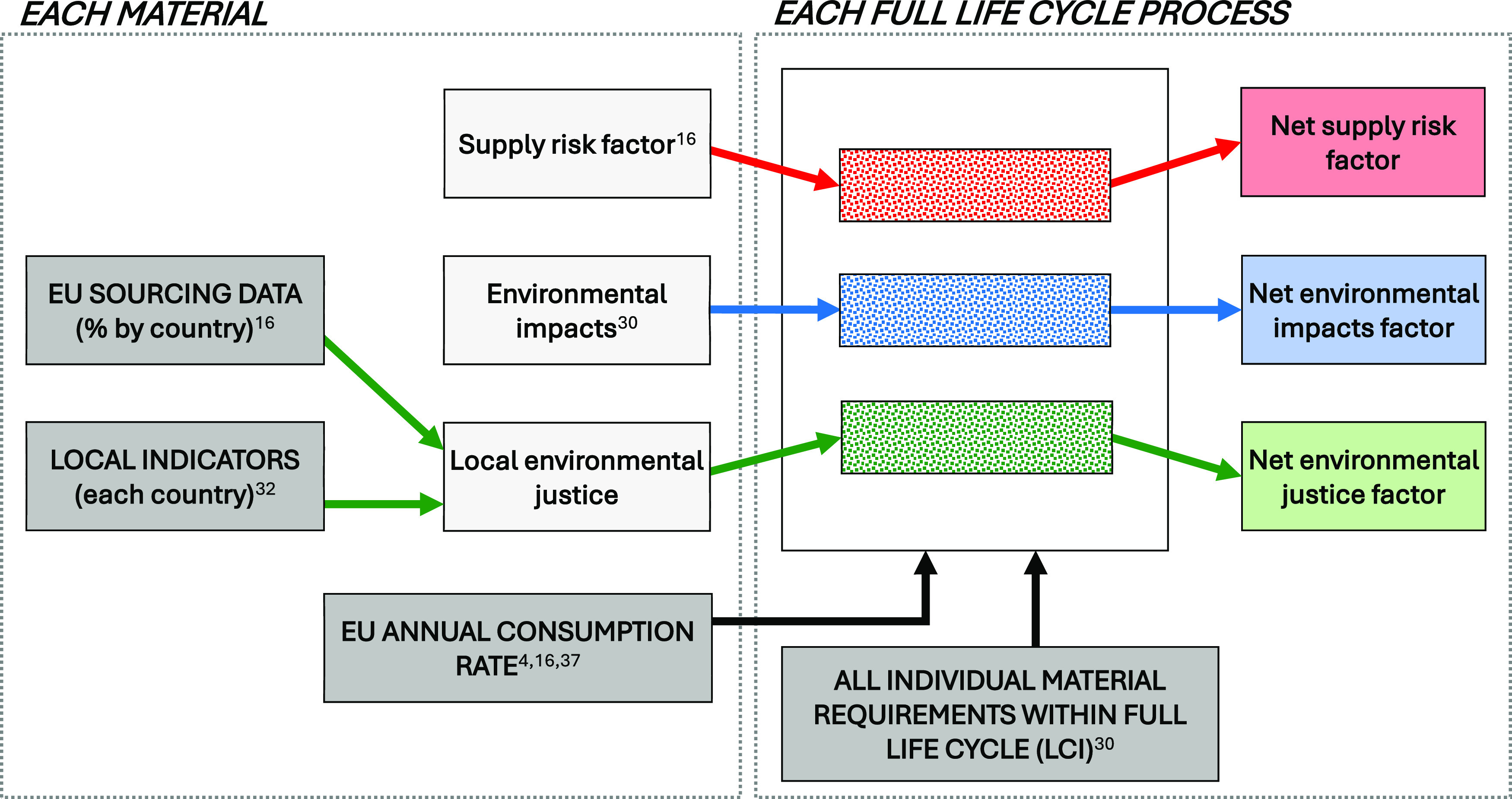
Conceptual overview of methods used for deriving the three raw material indicators for the extraction and processing of 55 selected materials. It is noted that all three indicators relate solely to the activities involved in deriving and supplying a specific group of raw materials to a process and do not attempt to quantify all supply risk, environmental impacts, or environmental justice aspects relating to the entire life cycle of that process.

Material requirement data for a given process—relating to the 55 selected materials—is then used alongside other data for each material to create the three composite indicator values. That is, individual “scores” can be obtained for any process defined by an LCI. The three final scores then enable direct comparisons of raw material indicators to be made for different processes. However, though the approach fundamentally provides scores for unit processes, the obtained scores can also be upscaled to provide composite scores for entire systems of individual processes. For example, in an energy system, each indicator can be applied pro-rata according to the relative contributions of each energy source to obtain composite scores that allow complete system configurations, such as those derived from energy systems modeling, to be compared. In this manner, the raw material characteristics of current and proposed energy systems can be analyzed for energy policy and planning purposes. Furthermore, as the methods are generically based on LCI definitions, it can equally be applied to any process defined within existing LCI databases. Figure 1 provides a final conceptual overview of the proposed approach prior to the detailed descriptions of each method.

### Supply Risk

The first method attempts to quantify the level of supply risk inherent to the sourcing of raw materials for a given process. Alongside an indicator of economic importance, the EC uses a derived measure of supply risk (SR) as one of the two key inputs in its own assessments of CRM status.^25,35^ In essence, the EC’s SR factor quantifies the potential risk of a disruption occurring within the supply chain of materials by considering the current sourcing locations of supplies and the governance and trade attributes of those locations. It is derived using the latest (2020) data for overall EU import reliance, a circularity indicator (the end-of-life recycling input rate), a substitution index, and two versions of the Herfindahl–Hirschman Index (HHI)^36^—derived using Worldwide Governance Index (WGI)^32^ data—that reflect the locational concentration and governance issues for the countries supplying the material at both EU and global levels. A complete listing of raw SR factor values for the materials examined in the study is provided in the Supporting Information.

Here, a composite SR indicator for a particular process is created by summing all SR factor values in proportion to the amount of the corresponding material required (mass) to produce one unit of the final “product” defined by the LCI. Initial attempts at deriving the indicator considered only these two inputs. However, it was soon discovered that using “raw” values of material requirement placed a large bias on materials used in larger amounts; this tended to vastly overshadow the significance of scarce materials used in much smaller amounts. For example, although both are considered to be CRMs, the required and available masses of materials such as silicon or titanium can be up to 5 orders of magnitude higher than those of rare earth materials. To overcome this bias, EU annual consumption levels^4,16,37^ were used as a “scaling” measure to represent the relative magnitudes of the requirements for different materials in the EU. As such, each material requirement value was first normalized by being divided by the corresponding EU consumption rate. Accordingly, the proposed formula for calculating the net SR factor for a given process is as follows
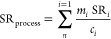
where, SR_process_ = net supply risk factor for the process under study [yr/MJ], *n* = number of selected individual materials in the process under study, *m*_*i*_ = mass of material *i* required by the process under study [kg/MJ], SR_*i*_ = supply risk factor of material *i* [dimensionless], and *c*_*i*_ = annual consumption level in EU of material *i* [kg/yr].

It is noted that, while the final value for the net SR factor is essentially dimensionless, the final units are actually the time frame of the consumption data divided by the unit that the material intensity is based upon—in this case, the relatively meaningless years per megajoule. Although calculations could also be undertaken using LCI data for processes based on different “functional units”—for example, megawatts of installed capacity or kilometers of travel—these would naturally return final values in different units. Though this demonstrates the flexibility of the method, it follows that one cannot directly compare final scores based on different functional units or consumption data.

### Local Environmental Impacts

A second method was developed to capture the potential for local environmental damages to occur during the extraction and processing of primary materials for a given process. Here, we once again rely on LCA data from the Ecoinvent database.^30^ However, in this instance, we follow the methodological guidance of Graedel et al.^38^ by utilizing LCIA endpoint indicators for the production processes of individual materials. As in this study, dimensionless indicators are derived for both ecosystem quality and human health for the production of a single kilogram of each material in accordance with the ReCiPe Endpoint (H,A) method.^31^

The ecosystem quality indicator aggregates values for terrestrial acidification, terrestrial ecotoxicity, freshwater ecotoxicity, marine ecotoxicity, freshwater eutrophication, agricultural land occupation, urban land occupation, natural land transformation, and climate change (on ecosystems). Analysis of the data suggests that this indicator is overwhelmingly influenced by some combination of marine ecotoxicity, natural land transformation, and climate change values for all materials examined. Meanwhile, the human health indicator aggregates values for human toxicity, photochemical oxidant formation, particulate matter formation, ionizing radiation, ozone depletion, and climate change (on human health). In this case, the indicator is overwhelmingly influenced by human toxicity values for all materials. A simple average of the net ecosystem quality and human health indicators was used as the final environmental impact (EI) value for each material. Although it is acknowledged that these impacts could occur anywhere along the supply chain of these raw materials, it is assumed here that a significant amount is directly related to the extraction and processing operations that occur near or close to their source locations. A full listing of the processes, LCIA endpoint indicators, and final composite EI values for each material are provided in the Supporting Information. It is noted that the values for gold and the three platinum group metals (PGMs)—palladium, platinum, and rhodium—are orders of magnitude higher than most of the other materials tested. This can be traced primarily to extremely high impacts encountered specifically during the extraction and refinery operations relating to these metals.^30^

The values for individual materials are then used to create a final indicator for a given process as follows
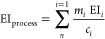
where, EI_process_ = net local environmental impacts score for the process under study [yr/MJ], *n* = number of selected individual materials in the process under study, *m*_*i*_ = mass of material *i* required by the process under study [kg/MJ], EI_*i*_ = local environmental impacts score for material *i* [dimensionless], and *c*_*i*_ = annual consumption level in EU of material *i* [kg/yr].

### Local Environmental Justice

A third method adopts a similar approach, this time attempting to determine how (un)just the sourcing of raw materials is likely to be for a given process. Though perhaps less directly tangible than SR and EI, the environmental justice (EJ) indicator seeks to widely embody a set of concepts that includes conflicts relating to the effects of pollution and the distribution of environmental risks.^39^ While the energy transition is widely predicted to exacerbate such issues at the global scale,^40,41^ much of the existing discourse on “energy justice” is focused on the siting of new facilities and the extraction and mining of fuels^42−47^ or on the embodied impacts caused by outsourcing energy, products, and services from other countries.^48^ In addition to this, a small number of previous studies have attempted to broadly address environmental justice issues in relation to the new infrastructure required to implement the energy transition.^26,49^ Meanwhile, a growing number of studies are attempting to quantify^27,28^ or catalogue^50^ justice-related issues specifically in relation to resource extraction and processing. Moreover, the burgeoning field of social life cycle assessment (sLCA) is beginning to address the impacts caused within these stages, including those used in energy production and in the new renewable energy infrastructure in particular.^23^ Nevertheless, to date, no studies have quantified justice elements in relation to specific materials or processes.

Here, we once again utilize information from an established dataset as a proxy indicator within the method. In this case, a composite value has been derived for each material using values taken directly from the Worldwide Governance Indicator (WGI) dataset,^32,33^ as used within the EC’s derivation of supply risk factor.^16^ The WGI provides values by country across six categories: voice and accountability, political stability and absence of violence/terrorism, government effectiveness, regulatory quality, the rule of law, and control of corruption. All six categories are thought to be generally associated with conditions that enable or reflect the potential for environmental justice issues to occur and, hence, are assumed to provide a suitable proxy for the potential occurrence of such issues. However, as the scores are provided on an arbitrary scale that typically ranges from around −2.5 to +2.5—where negative scores denote less desirable conditions and positive scores denote more desirable conditions—values for each indicator and each country are first normalized to percentage scores according to the range observed across all countries in that category. Accordingly, the proposed formula for calculating normalized composite WGI scores that equally weigh each indicator for each country is as follows
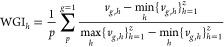
where, WGI_*h*_ = composite WGI indicator for country *h* [%], *p* = number of individual indicator categories in WGI database, *v*_*g*,*h*_ = value of indicator number *g* for country *h* [dimensionless], *z* = number of individual countries in WGI database.

Composite EJ indicators for each material are calculated by combining the WGI scores for each country and the percentage breakdown of global supply sources for each of the 80 candidate materials. However, as higher WGI scores reflect better environmental health characteristics, the values used are inverted by subtracting them from unity. Accordingly, the proposed formula for calculating the net environmental justice indicator for a given material is as follows
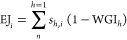
where, EJ_*i*_ = local environmental justice score for material *i* [dimensionless], *n* = number of countries included in analysis, *s*_*h*,*i*_ = share of global supply of material *i* sourced from country *h* [%], WGI_*h*_ = composite WGI indicator for country *h* [%].

As with the previous indicators, the composite EJ values for each material—as listed in the Supporting Information—can then be used to create a final indicator for a given process, viz.,
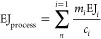
where, EJ_process_ = local environmental justice score for the process under study [yr/MJ], *n* = number of selected individual materials in the process under study, *m*_*i*_ = mass of material *i* required by the process under study [kg/MJ], EJ_*i*_ = local environmental justice score for material *i* [dimensionless], *c*_*i*_ = annual consumption level in EU of material *i* [kg/yr].

### Possible Applications

Calculating values of the three indicators for individual life cycle processes allows comparisons of different technologies or sub-technologies to be undertaken. For example, the indicators derived for a unit of heat or electricity from non-renewable sources could be directly compared with various renewable sources. Likewise, results for different sub-technologies could be compared within a technology group such as wind turbines or solar photovoltaic (PV) panels. Moreover, while the present article focuses on energy-related applications of the methods, it could theoretically be applied to any process defined by an LCI.

At a wider scale, scores for entire systems can be generated by tallying the product of the indicator and the total energy generated by each technology to derive final systemwide values. This would enable, for example, the characteristics of current systems to be compared against multiple future alternatives to inform policy decision-making. The proposed formula for calculating aggregated scores over entire systems is as follows
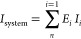
where, *I*_system_ = aggregated indicator score for the system under study, *n* = number of selected individual processes in the system under study, *E*_*i*_ = total energy production derived from technology, and *iI*_*i*_ = indicator score of the process *i*.

## Case Study: EU Electricity Supply

To demonstrate the value and functionality of the proposed methods, they are applied here to an exploratory case study involving existing and projected electricity generation levels for the EU, by technology, according to the EC’s latest “reference scenario.”^51^ Values are firstly derived for all available individual LCI listings within the Ecoinvent database.^30^ Using mean values for each technological group defined within the EC data, aggregated system values were then derived using values from the EC scenario data sets to determine predicted changes in the three indicators under these assumptions.

### Individual and Grouped Scores by Technology

**Figure 2:**
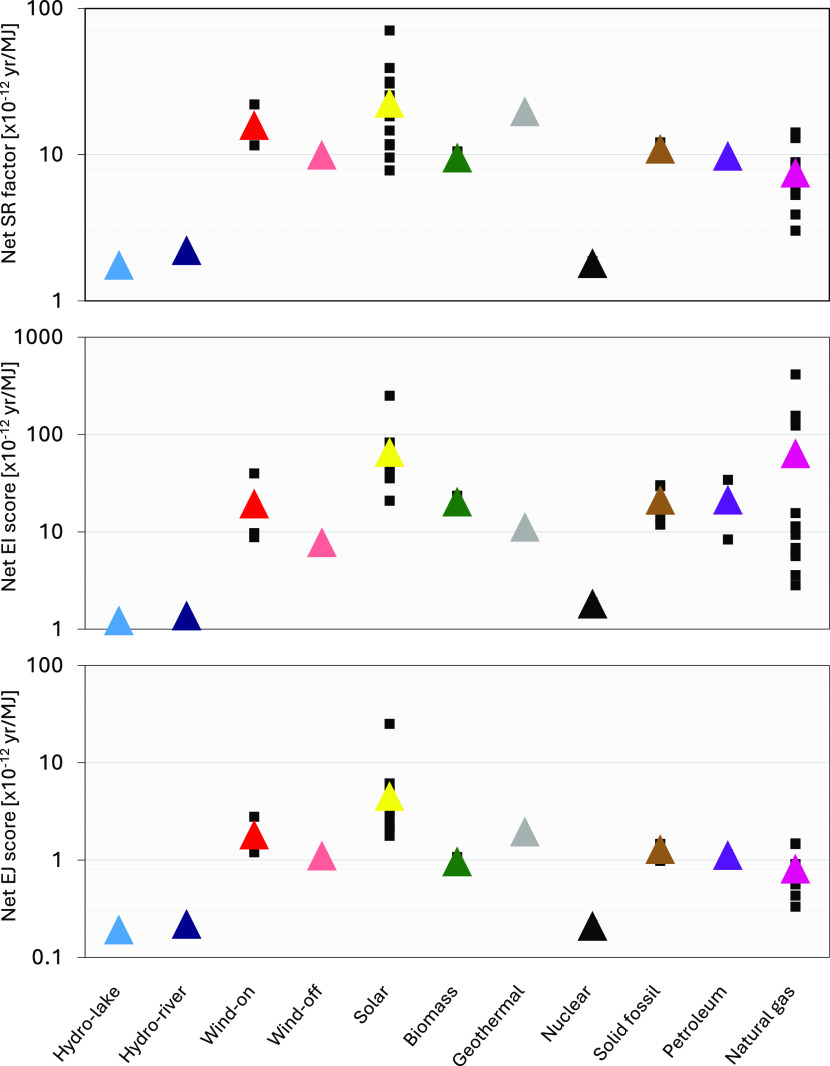
Results for supply risk factors and environmental impacts and environmental justice scores for all available processes, grouped by category. Mean values shown as colored triangles.

Using the 11 technological categories defined within the reference scenario as a basis, all 51 regionally applicable electricity production processes within the 2021 version of the Ecoinvent LCI database^30^ were collected and grouped. Values of the three indicators were then derived for each individual process on a per-MJ basis as displayed in Figure 2. A full listing of the inputs and results is supplied in the Supporting Information.

The results for the three indicators demonstrate a relatively clear pattern across all three methods. The mean results by category suggest that risks and impacts are considerably lower for lake and river hydropower and nuclear processes, reflecting their relative simplicity and lower reliance on CRMs. Values for the three fossil fuel sources—natural gas, petroleum, and solid fossil—are typically moderate, although natural gas scores are generally lower for SR and EJ. Notwithstanding this, major variations are observed for natural gas in the EI category, where three of the 12 processes are significantly higher as a result of their high reliance on platinum and rhodium; all other natural gas processes are far more consistent with scores observed for the other two indicators.

Values for biomass sources also tend to be in this moderate range alongside offshore wind turbines, although the value for offshore wind is somewhat lower in the EI category. Conversely, the scores derived for onshore wind are approximately double these levels as a result of their elevated reliance on rare earth materials, predominantly in the permanent magnets used in certain generator mechanisms;^52−54^ the offshore turbine assessment within the Ecoinvent data set assumes the use of hybrid approaches that rely less on rare earth materials. Values for geothermal energy are high in the SR and EJ categories but are noticeably lower for EI and are only considered moderate.

The solar technologies group—which includes both PV panels and concentrated solar power (CSP) plants—is more extensive than other categories, reflecting the many different approaches employed in the field. Values for different solar technologies range from moderate to very high and more or less cover an entire order of magnitude for each indicator. Copper indium gallium selenide (CIS) cells represent the higher scores in all three indicators, largely based on a strong need for gallium.

The relatively consistent trend observed in the results reflects the influence of using the same masses of material (*m*_*i*_)—derived from LCI listings—across all three sets of calculations. As such, each indicator can be seen to be, first and foremost, a reflection of the total amount of all key materials required—relative to total consumption—per unit of output; a process that uses higher levels of key materials overall will always be more likely to obtain higher scores than those with lower material requirements. In this sense, while the overall trends are clear cut, the three inputs applied for each material—SR_*i*_, EI_*i*_, and EJ_*i*_—can be viewed as contributing varying levels of additional “scaling” within each calculation.

Nevertheless, variability in these “scaling” inputs can still be influential and result in significant variations in indicator results, particularly where inputs are not well correlated for a given set of materials. This issue is further investigated via a series of regression analyses, provided in the Supporting Information. Regression analyses on the three indicators at the material and process level revealed that the results appear to be suitably “unique” at the material level, particularly for EI values that are significantly different from the findings for SR and EJ. Notably, despite the fact that both include data from the WGI database in their derivation, the “*R*-squared” (*R*^2^) value comparing SR and EJ at the material level was found to be relatively low (0.15780). In any case, the common materials use amounts used in both calculations scale up these factors and provide similarity at the process level.

### Current and Projected Scores for the EU System

To demonstrate the application of the three indicators to real-world scenarios, they are applied to projected values of gross electricity generation, by source, from the EU reference scenario;^51^ observed and projected values for the 11 categories are provided at five-year intervals from 2005 to 2050, as listed and illustrated in the Supporting Information. The raw data demonstrate that wind, solar, and biomass are the only technologies to have risen significantly since 2005 although this trend is not expected to continue for biomass. Although geothermal sources are expected to rise slightly after 2035, the utilization of onshore and offshore wind and solar technologies are projected to increase by factors of 2.9, 7.6, and 4.2, respectively, between 2020 and 2050. All other technologies are seen to remain relatively stable going forward. However, in the cases of petroleum and solid fossil fuels, levels are predicted to decrease by factors of 30.2 and 9.6, respectively. As such, wind, solar, petroleum, and solid fossil fuels are expected to have the biggest influence on overall changes across all three factors.

**Figure 3:**
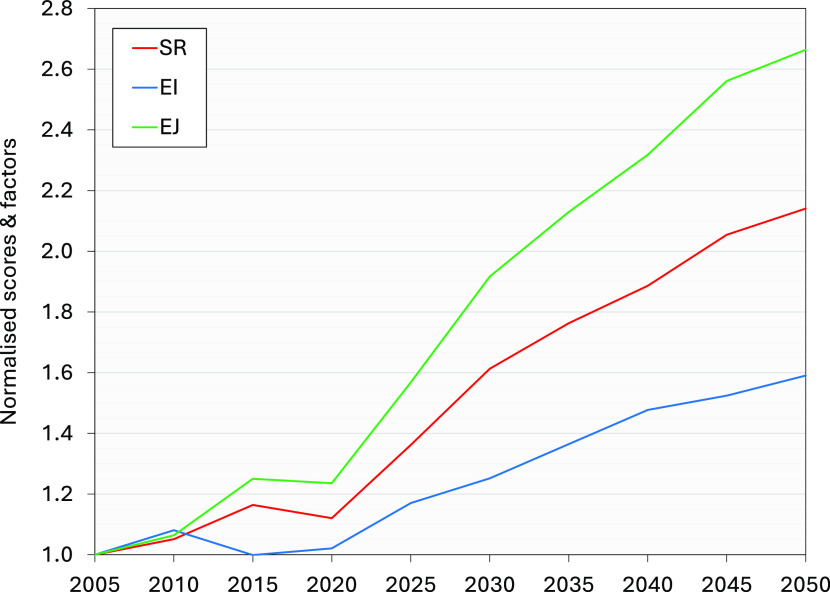
Normalized values of overall supply risk (SR) factors and environmental impacts (EI) and environmental justice (EJ) scores for the EU electricity system for the period 2005 to 2050 according to technological projections defined by the EU reference scenario.^51^ All values are relative to the dimensionless values of SR, EI, and EJ for 2005 of 67.8, 219.8, and 7.7, respectively. It is noted that the projections are based on the current values for SR, EI, and EJ for each material. As such, they do not purport to predict any future variations in these factors for different materials going forward or to reflect previous values. Rather they are used to broadly illustrate the vulnerability of forecast energy systems in the EU to raw material supply issues based on current estimates for each material.

Values for individual technological categories are first calculated by multiplying the mean values for each indicator—in yr/MJ—by the amount of energy reported for that category—in MJ/yr—in the EU data. Final system values for the three indicators are then calculated by aggregating the scores for all 11 categories. Final values for the three indicators at each interval—normalized to “base” levels in 2005—are shown in Figure 3; a full listing of these results is also provided in the Supporting Information.

The results indicate that the overall values of each indicator are likely to increase dramatically under the forecast scenario. Values of SR and EJ are both predicted to more or less double between 2020 and 2050; the value of EI is expected to rise by around 56%. A small downturn was noted in the SR and EJ observations between 2015 and 2020, largely due to the especially hurried withdrawal of solid fossil fuels and petroleum—which have significantly higher per-MJ values for both SR and EJ than legacy non-renewable technologies like natural gas and nuclear—and the slight reduction in the growth rate of the highest-ranking group, solar. Similarly, a drop in EI values between 2010 and 2020 is predominantly the result of a drop in the use of natural gas, which has the second-highest per-MJ value for EI. A full set of results is provided in the Supporting Information.

It is noted that these calculations do not take future variations in SR, EI, and EJ inputs into account. Present-day values are assumed when, in reality, these values are likely to fluctuate over time because of geopolitical shifts, technical advancements, changes in recycling practices, or discoveries of new reserves. Nevertheless, this example provides a simple demonstration of the potential issues that could result from transitioning to renewable technologies that will most likely continue to rely on materials with higher risk factors.

In the end, the key finding here is that the substantial rises in electricity from wind and solar sources that are predicted by 2050 look likely to result in significant increases in the net scores for all three of the examined indicators. Indeed, onshore wind and solar technologies are predicted to generate the highest and third-highest amounts of electricity, respectively, by 2050 while also representing the third-highest and highest per-MJ scores for each of the indicators.

### Sensitivity Analysis

While using annual EU consumption values, *c*_*i*_, appears to be a logical way to normalize scores and avoid issues of disproportionate weighing in the presented methods, it was deemed necessary to test the influence of these values on final scores. To do this, a simple sensitivity analysis was undertaken. To allow for uncertainties in the estimates of *c*_*i*_, an additional 20% was added to the annual consumption values for a group of 13 key materials, all of which are highly influential in determining indicator values while having annual consumption rates of less than 1,000 tonnes. As expected, all three indicators were shown to be sensitive to these changes, with reductions of between 11.7 and 16.6% being observed. However, very low standard deviations—between 1.0 and 2.8%—were observed within the changes, suggesting that the method can maintain consistent delineation between processes when uncertainties in inputs are experienced. Full details of the analysis are contained in the Supporting Information.

## Discussion

Raw material supply is an ongoing concern in relation to the transition to renewable energy sources. Although we are limited to present-day assumptions about material supply characteristics, applying newly developed methods to EU system projections strongly suggests that the potential for environmental impacts and justice issues to occur during the extraction and processing phases of the identified set of key materials looks likely to rise dramatically over the coming decades. Likewise, the overall risks associated with obtaining these materials also look set to increase sharply based on current projections. Recent disruptive events such as the COVID-19 pandemic and war in Ukraine have highlighted the fragility of global markets to supply chain issues and made the consequences of such disruptions more tangible in the minds of many. Indeed, Russia currently produces 33 of the 44 materials identified as CRMs by the EC;^16^ for five of these—palladium (40.0%), scandium (26.0%), titanium (22.0%), platinum (12.9%), and rhodium (12.0%)—Russia supplies over 10% of current global supplies.

Meanwhile, China is a known producer of 39 of these 44 materials and is responsible for over 80% of current global supplies of 16 such materials, including gallium, germanium, and all light and heavy rare earth metals, all of which are important in the manufacture of wind turbines and solar PV panels.^4^ Ongoing tensions between China and the west could have very serious implications in this regard.^52,55,56^ For certain materials, increased levels of recycling could help offset strong import reliances, although recycling activities would also need to be undertaken at the local level to avoid further supply-related issues relating to the importation of recycled materials. Either way, circularity principles look likely to become an integral part of future raw material landscapes.^57,58^ Nevertheless, many CRMs are technically difficult to recover from waste streams^25^ and strong reliance on newly extracted materials looks set to continue for the foreseeable future. Collectively, these observations highlight the need to continue to monitor key materials and to assess the indicators that best reflect the status of these materials over time.

In any case, while most discussions in this area concern the locations of global reserves and the importance of maintaining adequate supply lines, localized environmental impacts during the material extraction and processing stages, and aspects of environmental justice that relate to these impacts, are increasingly being considered. The methods introduced here represent a first attempt at addressing this gap. Furthermore, as the three methods are fundamentally based on listings of individual materials required to produce one “unit” of a given process, they could theoretically be applied to any process defined by an LCI listing and could, theoretically, find use in any number of applications inside and outside of the energy sphere.

Results from the case study strongly suggest that renewable technologies within the wind, solar, and geothermal categories present higher SR, EI, and EJ values than other technologies, while fossil fuel technologies tend to present midrange values. The higher scores for solar and wind energy present a particular cause for concern in this regard, especially when coupled with the fact that both technologies are expected to play key roles in most predicted transition scenarios worldwide.^51,59,60^ While continuing to rely on fossil fuels would result in lower scores in all three indicators, other ramifications relating to these technologies—not least of which are far higher GHG emissions—mean that they are generally no longer considered viable future alternatives. Conversely, although hydropower, biomass, and nuclear technologies also bring their own constraints and controversies, it is noted that their potential to introduce disturbances is among the lowest in all three metrics considered here. At any rate, it is hoped that the methods and findings presented will further highlight the seriousness of raw material issues in energy transition processes and the need to interrogate and balance these aspects when considering different technological options.

Nonetheless, while these approaches are thought to represent an original and valuable contribution to the field, several limitations are noted. First, they only consider the group of 80 materials identified as potentially critical by the EC.^16^ As such, other key materials could potentially be neglected for certain processes, and aspects relating to the extraction and processing of fossil fuels and uranium—particularly in relation to localized environmental impacts and justice issues—are not included. Furthermore, 25 of the 80 identified materials are not currently represented in the LCI databases. Again, though many of the omitted materials are not considered vital, materials such as niobium, germanium, and indium are known to be important in a number of key future technologies.^4,6,49,61,62^ Wider inclusion of materials in future LCI data releases would provide more robust coverage in this respect.

Similarly, many key technologies are poorly represented in current LCI data sets, limiting deeper analyses or comparisons. For example, only one type of geothermal electricity and two types of solar CSP are represented, and listings for key renewable energy technologies such as biofuel production, power-to-gas (P2G), power-to-liquids (P2L), hydrogen electrolysis, and most forms of electrical storage are almost entirely absent in the current databases. Wind power, widely predicted to be a dominant player in most future energy scenarios, is only represented by three onshore processes and one offshore process in the latest Ecoinvent database compared to the 19 listings for solar technologies. Although data can sometimes be obtained from secondary sources,^63^ more complete listings of key technologies within universal databases such as Ecoinvent^64^ and GaBi^65^ would greatly improve the ability of practitioners to assess future energy systems.

It is also important to address locational issues as they relate to the methods being presented. As the SR factors being used were specifically derived for EU supplies, they can strictly only be used for processes occurring within the EU. Naturally, local SR factors could be vastly different in certain countries, particularly in those that are dominant suppliers of particular materials or use different supply mixes. Conversely, the calculations for EI and EJ are far more universal as they rely on global supply mix data or LCIA data where only a single global estimate is used. This highlights the fact that the SR method intrinsically assumes that SR is the same whether materials are brought to the EU as raw and processed materials or embedded within intermediate products; this is thought to be an acceptable assumption in lieu of vastly more complex calculations. Likewise, owing to the complex array of components within most products and processes, it is assumed that using global data is suitable when assessing EI and EJ scores.

Nevertheless, higher levels of granularity in LCA data sets, particularly in relation to the locations in which sub-processes occur, would allow more complete assessments of intermediate materials, components, and finished assemblies to be undertaken. In this regard, future studies could attempt more-detailed assessments involving sub-processes within overall processes. As many such sub-processes are likely to occur outside of the EU, SR factors would need to be calculated for each material for different regional locations using a similar approach to that used in the EU.^16^ For example, SR factors in China would be vastly different for materials they are currently key suppliers of and a sub-process occurring in China would then need to use these inputs. The same is true for calculating EJ scores in different territories, where specific supply mixes could theoretically be applied, and EI scores could use more specific LCA processes for materials where regional data exist. Such assessments would be large undertakings and are well beyond the boundaries of the current study. However, the concept could provide a basis for future research.

The supply of raw materials looks likely to remain a concern as we attempt to implement greater levels of renewable energy and other strategic technologies going forward. As such, robust methods for quantifying the constraints and other aspects relating to raw material supply are vital to ensuring that decarbonization pathways are optimized at all levels. In this sense, it is hoped that the introduced methods provide a valuable new contribution to the field of raw material supply at large and a specific starting point for energy modeling and related applications as we strive to optimize pathways toward more sustainable energy systems.

## Supporting Information Available

Description and results of validation and sensitivity analysis (PDF)
Full listings of data used in analysis (XLSX)
