# Supplementary material for: New Techniques for Assessing Critical Raw Material Aspects in Energy and Other Technologies
Source: Environ Sci Technol. 2022 Nov 24;56(23):17236–45. doi: 10.1021/acs.est.2c05308 (PMC9730842; doi:10.1021/acs.est.2c05308)
Supplement: Supplementary file 1 — es2c05308_si_001.pdf [file es2c05308_si_001.pdf]

New Techniques for Assessing Critical Raw Material Aspects in Energy and Other Technologies

Nick Martin[1,], Cristina Madrid-López[1], Gara Villalba Méndez,[1,2] and Laura Talens Peiro[1]*

* nicholas.martin@uab.cat

1 Sostenipra Research Group, Institute of Environmental Science and Technology (ICTA-UAB), Autonomous University of Barcelona, Bellaterra (Cerdanyola del Vallès), Catalunya, 08193, Spain

2 Department of Chemical, Biological and Environmental Engineering, Autonomous University of Barcelona, Bellaterra (Cerdanyola del Vallès), Catalunya, 08193, Spain

10 pages

Figure S1. Results for net supply risk (SR) factors by technological category: (a) base results, (b) sensitivity results for increasing consumption values for 13 key materials by 20%
Figure S2. Results for net local environmental impacts (EI) scores by technological category: (a) base results, (b) sensitivity results for increasing consumption values for 13 key materials by 20%

Figure S3. Results for net local environmental justice (EJ) scores by technological category: (a) base results, (b) sensitivity results for increasing consumption values for 13 key materials by 20%

Table S1. Summary of regression analysis results. Correlations analyzed between derived SR, EI and EJ values for individual materials and final SR, EI and EJ values for complete processes

Table S2. Summary of materials with highest contributions to indicator scores for electricity technologies, in order of mean supply risk (SR) factor contribution

Table S3. Summary of sensitivity analysis for electricity technology categories

FURTHER ANALYSIS

Two further processes of investigation were undertaken to validate and test the sensitivity of the case study results. Firstly, a regression analysis was undertaken to test the levels of independence in the data at the material and process levels. Secondly, a sensitivity analysis was undertaken to test the effect that changes in one of the input parameters–EU consumption levels, a denominator in the calculations for specific technologies–could have on the final results.

Regression analysis. In order to test the uniqueness of the three methods, a series of regression analyses were undertaken to determine the levels of correlation that exist between the data at the different stages of the overall approach. Analyses were performed on the intermediate results for supply risk (SR), environmental impact (EI) and environmental justice (EJ) indicators for the individual *materials* selected and the final SR, EI and EJ indicators for all 51 of the electricity production *processes* examined in the study. A simple least-squares regression analysis was performed on the combinations of indicators at each level. "R-squared" (R^2) values were chosen as the most appropriate indicator for the analysis. The results are displayed in Table S1.

Table S1. Summary of regression analysis results. Correlations analyzed between derived SR, EI and EJ values for individual materials and final SR, EI and EJ values for complete processes

	Variables	R-squared value
For materials	SR, EI	0.00319
	SR, EJ	0.15780
	EI, EJ	0.00114
For processes	SR, EI	0.15857
	SR, EJ	0.83447
	EI, EJ	0.20772

Firstly, the derived values of SR, EI and EJ for individual *materials* were all found to be poorly correlated, particularly when comparing EI with SR and EJ, which returned final R-squared values of 0.00319 and 0.00114, respectively. The value for the relationship between SR and EJ was found to be comparatively high–0.15780–confirming that there is a link between these two indicators, most likely derived from their mutual use of inputs from the World Governance Index (WGI) database[1]. Nevertheless, all of these connections should ultimately be considered to be low. This highlights the relative "uniqueness" of the three indicators at the material level.

Secondly, when SR, EI and EJ values for individual materials are upscaled for selected *processes*–according to the amounts of each material stated in the life cycle inventory (LCI) listings obtained from the Ecoinvent database[2]–higher levels of correlation are observed. This is to be expected in such cases, when all values are "scaled-up" using the same material use amounts. Here, SR is shown to be well correlated to EJ, with an R^2 value of 0.83447. The findings for EI to SR and EI to EJ–0.15857 and 0.20772, respectively–suggest that some correlation is observed, largely as a result of the common material use amounts used in the calculations for different LCA processes. In all, the regression analysis confirms that the three factors are all suitably "unique" at the *material* level but that the common material use amounts scale up these factors and provide at least some similarity at the *process* level.

Sensitivity to changes in annual consumption values. In order to test the validity of the analysis and determine the sensitivity of the results to changes in input parameters a test was performed on the denominator used in each of the final indicator calculations, the existing levels of consumption within the EU. To do this, it was first necessary to assess the relative contributions of individual materials to the final scores for the indicators in order to detect the most "influential" and, hence, suitable, materials for the test. This was achieved by calculating the percentage contributions of

each material to the final indicator scores for each of the Ecoinvent processes analyzed in the case study for the EU electricity system.

Table S2 displays the mean and maximum contributions of the 20 most significant materials to each of the indicators. The results show that a group of critical raw materials (CRMs) with relatively low annual consumption rates–including gallium and several light (LRE) and heavy (HRE) rare earth materials–tend to dominate the scores for these indicators. It is interesting to note that gold and the three platinum group metals (PGMs) included here–platinum, rhodium and palladium–were found to make far higher contributions to EI scores on account of the high environmental impacts that relate to their respective extraction activities. The data for maximum contributions also demonstrates the significance of certain materials in specific processes, the best example being gallium and its overwhelming impact on the values for copper indium gallium selenide (CIS) solar photovoltaic cells.

As the calculations for each indicator require individual contributions to be divided by the annual EU consumption amount, indicators are particularly sensitive to changes in these values. This is especially true as most of the influential materials are consumed in low amounts; low denominators, therefore, result in higher contributions. Accordingly, to test the sensitivity of the

calculations to uncertainties in the estimated annual EU consumption levels, 20% was added to the levels of all 14 of the 20 identified materials that have current annual consumption estimates under 1,000 tonnes. Updated results for this sensitivity scenario are shown for SR, EI and EJ in Figure S1(b), Figure S2(b) and Figure S3(b), respectively. A summary of the percentage changes that occur to the derived SR and EI values under this scenario are shown in Table S3. The figures and tabulated data suggest that, although the changes to the overall values of the two indicators are significant–between 11.7% and 16.6%–the changes are generally very consistent, with observed standard deviations for all indicators across all processes of between 1.0% and 2.8%.

Table S2. Summary of materials with highest contributions to indicator scores for electricity technologies, in order of mean supply risk (SR) factor contribution

				SR factor			EI score			EJ score		
Material	EU CRM?[3]	Rare earth/PGM?	Annual EU consumption[3–5]	Value[3]	Mean contribution	Max contribution	Value[2]	Mean contribution	Max contribution	Value[1]	Mean contribution	Max contribution
			[tonnes]		*[%]*	*[%]*		*[%]*	*[%]*		*[%]*	*[%]*
Samarium	x	LRE	6	6.12	28.2%	33.5%	1.13	3.9%	10.7%	0.51	19.3%	28.1%
Neodymium	x	LRE	100	6.07	21.3%	24.7%	2.76	7.1%	19.8%	0.51	14.6%	21.0%

Praseodymium	x	LRE	41	5.49	15.3%	17.8%	3.98	8.2%	22.7%	0.51	11.6%	16.7%
Gallium	x		27	1.26	7.8%	83.2%	4.81	10.9%	89.6%	0.50	16.0%	93.1%
Rhodium	x	PGM	7	2.14	0.1%	1.0%	6,240	21.0%	50.7%	0.47	0.1%	1.9%
Gadolinium	x	HRE	11	6.06	7.9%	9.2%	5.12	4.9%	13.6%	0.51	5.4%	7.8%
Platinum	x	PGM	64	1.84	0.0%	0.7%	5,860	17.3%	40.4%	0.46	0.1%	1.6%
Lanthanum	x	LRE	645	6.04	6.9%	8.2%	2.06	1.7%	4.8%	0.51	4.8%	7.0%
Gold			1,425	0.19	0.0%	0.0%	3,501	11.8%	42.3%	0.49	0.0%	0.1%
Tellurium			30	0.51	1.2%	7.2%	0.59	0.6%	1.9%	0.43	5.9%	22.4%
Palladium	x	PGM	59	1.27	0.0%	0.4%	1,570	6.5%	17.2%	0.49	0.1%	1.2%
Magnesite			49,459	0.65	0.7%	1.6%	0.04	0.0%	0.1%	0.51	4.3%	10.6%
Baryte	x		506,410	1.26	0.8%	6.7%	0.15	0.1%	1.0%	0.54	3.2%	24.5%
Magnesium	x		113,000	3.91	1.7%	39.7%	1.87	0.6%	22.5%	0.52	1.7%	44.0%
Tantalum	x		395	1.36	0.9%	5.3%	1.99	0.4%	1.7%	0.64	2.1%	9.2%
Beryllium	x		38	2.29	0.4%	8.0%	29.62	2.3%	38.6%	0.34	0.5%	9.0%
Phosphorus	x		48,300	3.55	1.4%	13.6%	0.17	0.0%	0.2%	0.52	1.5%	15.0%
Dysprosium	x	HRE	14	6.20	1.3%	1.6%	0.06	0.0%	0.0%	0.51	0.9%	1.4%
Tungsten	x		431	1.61	0.4%	8.2%	4.11	0.5%	7.8%	0.52	1.0%	20.0%
Silver			3,800	0.68	0.1%	0.5%	36.97	1.4%	5.4%	0.38	0.2%	1.2%

As such, it is concluded that, while the results are certainly sensitive to changes in values of EU consumption for the most influential materials, the overall findings in the results are not altered in any significant way. These findings also reinforce the idea that, ultimately, the results are heavily influenced by the levels of individual material use in each process and confirms that small changes

to the parameters relating to those materials will not drastically alter the rankings for a set of processes.

Table S3. Summary of sensitivity analysis for electricity technology categories

Category	Mean percentage changes per category *[%]*		
	SR	EI	EJ
Hydro–lake	-15.7	-16.2	-13.9
Hydro–river	-15.9	-16.2	-14.1
Wind–onshore	-15.8	-13.7	-13.9
Wind–offshore	-15.6	-15.4	-13.2
Solar	-15.2	-12.0	-14.9
Biomass	-15.7	-16.2	-13.9
Geothermal	-15.8	-16.1	-14.0
Nuclear	-15.6	-15.7	-13.4
Solid fossil	-14.5	-16.3	-13.4
Petroleum	-14.9	-16.4	-11.7
Natural gas	-15.6	-16.6	-13.7

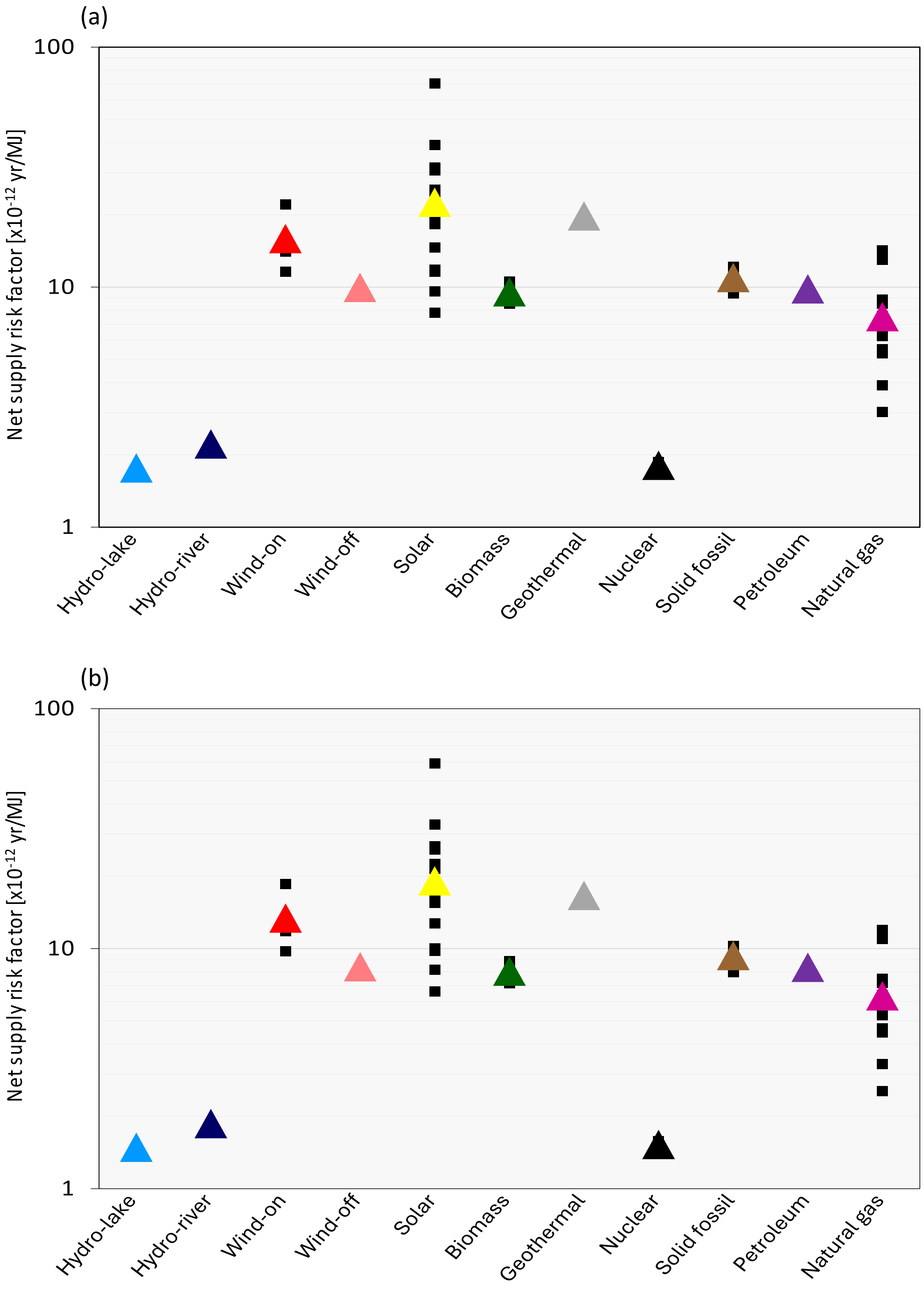

Figure S1. Results for net supply risk (SR) factors by technological category: (a) base results, (b)

sensitivity results for increasing consumption values for 13 key materials by 20%

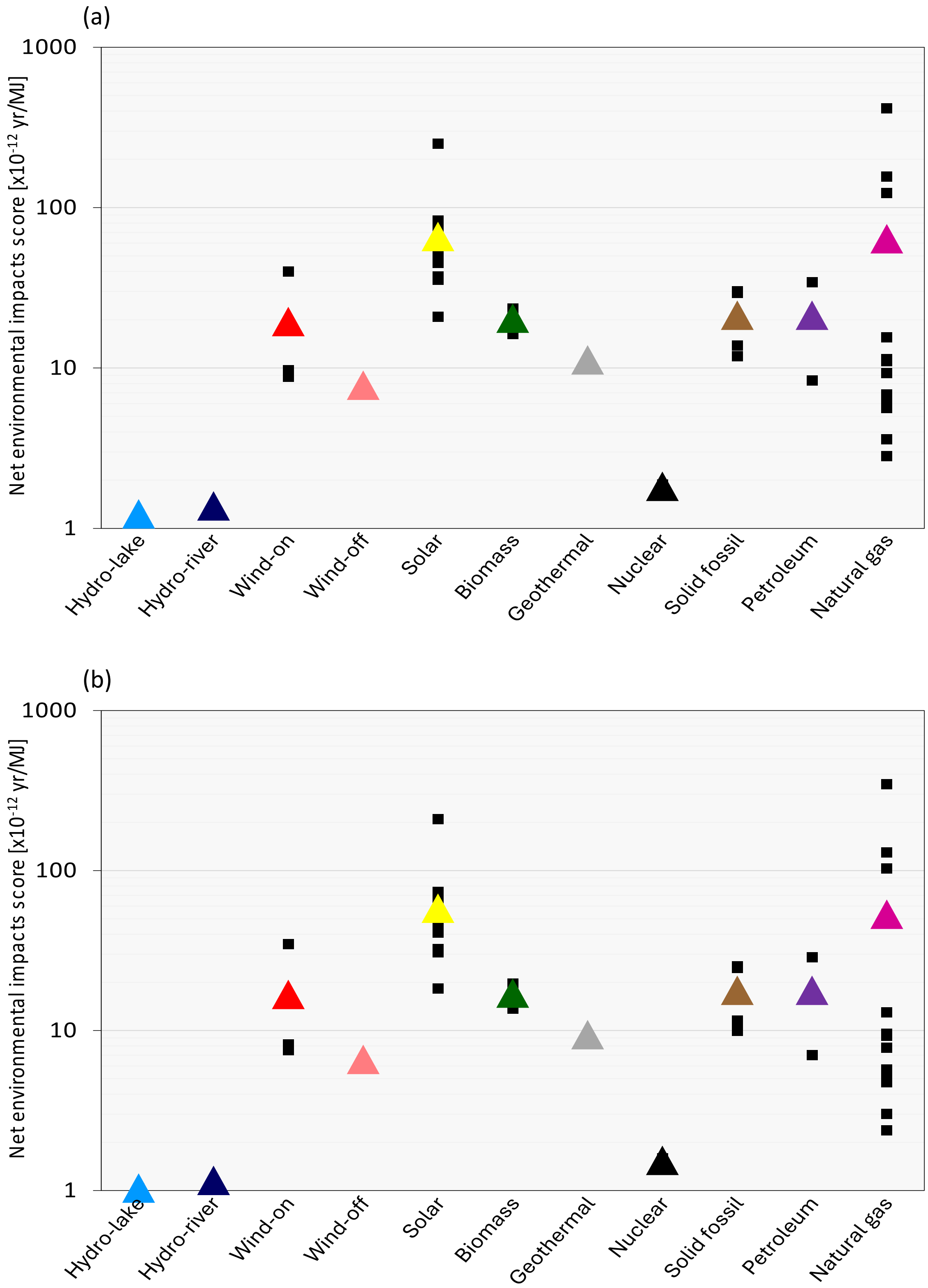

Figure S2. Results for net local environmental impacts (EI) scores by technological category: (a)

base results, (b) sensitivity results for increasing consumption values for 13 key materials by 20%

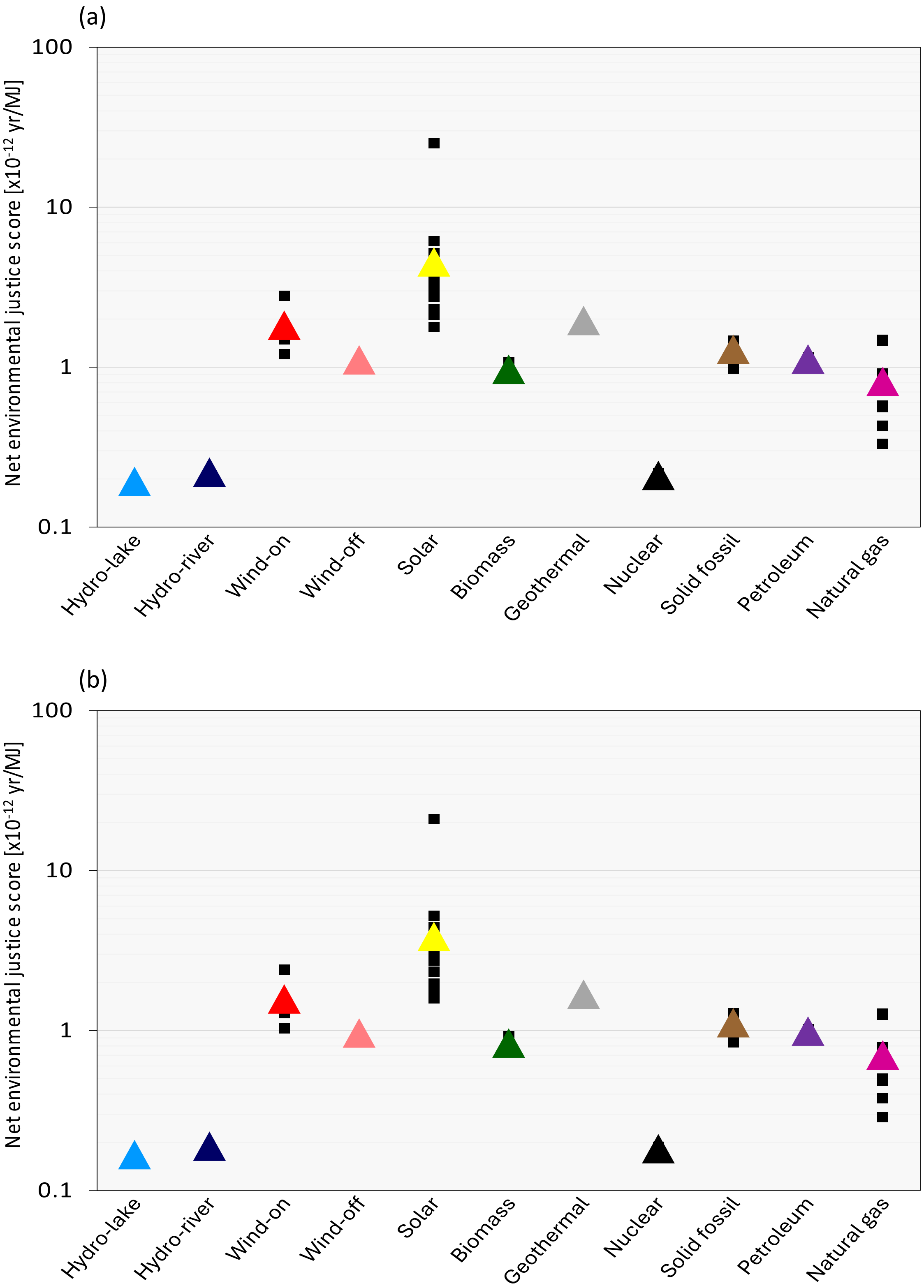

Figure S3. Results for net local environmental justice (EJ) scores by technological category: (a)

base results, (b) sensitivity results for increasing consumption values for 13 key materials by 20%

REFERENCES

(1) The World Bank. *Worldwide governance indicators: World Bank data catalog.* http://info.worldbank.org/governance/wgi/ (accessed 2022-06-27).

(2) Ecoinvent. *Ecoinvent version 3.8 (2021) database.* https://v38.ecoquery.ecoinvent.org/Home/Index (accessed 2021-10-05).

(3) European Commission. *Study on the EU's List of Critical Raw Materials (2020) - Final Report*; Publications Office of the European Union: Luxembourg, 2020. https://doi.org/10.2873/11619.

(4) Bobba, S.; Carrara, S.; Huisman, J.; Mathieux, F.; Pavel, C. *Critical Raw Materials for Strategic Technologies and Sectors in the EU: A Foresight Study*; Publications Office of the European Union: Luxembourg, 2020. https://doi.org/10.2873/865242.

(5) Eurostat. *EU trade since 1988 by HS2,4,6 and CN8 [DS-645593], Extra-EU28, IMPORT, QUANTITY_IN_100KG.* https://appsso.eurostat.ec.europa.eu/nui/show.do?dataset=DS-645593&lang=en (accessed 2022-07-30).
